# The Influence of Colostrum and WPC Preparations on the Quality Physicochemical, Functional and Sensory Parameters of Milk Fermented Drinks

**DOI:** 10.3390/foods15050919

**Published:** 2026-03-06

**Authors:** Marcelina Maciejewska, Marek Nowak, Anna Mandecka, Marek Szołtysik, Anna Dąbrowska

**Affiliations:** Department of Development Functional Food Products, Faculty of Biotechnology and Food Science, Wroclaw University of Environmental and Life Sciences, 50-366 Wroclaw, Poland; marek.nowak1@upwr.edu.pl (M.N.); anna.mandecka@upwr.edu.pl (A.M.); marek.szoltysik@upwr.edu.pl (M.S.); anna.dabrowska@upwr.edu.pl (A.D.)

**Keywords:** whey proteins, yogurt, probiotic viability, antioxidant capacity, rheology, syneresis, sensory

## Abstract

This study investigated the effects of bovine colostrum and whey protein concentrate (WPC) on the physicochemical, functional, microbiological, and sensory properties of fermented milk beverages formulated with different ingredient compositions and starter culture variants. Four formulations were evaluated during two weeks of refrigerated storage. WPC addition markedly reduced viscosity, with the lowest value observed in WPC enhanced samples (0.26 Pa·s), whereas skimmed milk powder contributed to a more balanced texture. Syneresis was highest in the WPC-rich formulation (6.9 mL) and lower in colostrum-containing samples (3.2–4.9 mL), indicating improved water-holding capacity. Colostrum enhanced antioxidant activity, with ABTS values reaching approximately 240–250 µM Trolox/mL during mid-storage, followed by a decline on day 14. Sensory evaluation showed the highest consumer acceptance for samples containing balanced proportions of colostrum and WPC, while formulations with high WPC content scored lower due to inferior texture and appearance. The applied formulations also supported the viability of *Bifidobacterium* spp. during refrigerated storage, maintaining counts at levels considered adequate for probiotic dairy products. Overall, the combined use of bovine colostrum and WPC resulted in fermented milk beverages with improved functional properties, structural stability, probiotic viability, and sensory acceptability.

## 1. Introduction

The market for functional foods has been expanding rapidly, driven by consumers’ increasing health awareness and interest in disease prevention [1,2,3]. Recent findings indicate that costumers willingness to pay more for products is mainly determined by health-related attributes [4]. Previous studies demonstrate that such products are most readily accepted when delivered through familiar channels, such as dairy, with fermented milks being perceived as natural and convenient [5,6]. These trends emphasize the potential of creating novel functional dairy products incorporating bioactive compounds.

Bovine colostrum, the initial secretion following parturition, is a nutrient-rich fluid characterized by its high protein, lipid, vitamin, mineral, and bioactive compound content, including immunoglobulins, lactoferrin, and growth factors [7,8]. The composition and biological activity of this substance vary across different species, and its functional potential is influenced by differences in protein, fat, antioxidant capacity, and mineral content [9,10]. Beyond its nutritional profile, colostrum provides a favorable environment for the growth of probiotic bacteria, which supports its application in fermented dairy products [11]. From a production perspective, dairy cows generally produce colostrum in volumes exceeding the nutritional requirements of calves, leaving a significant surplus that cannot be marketed as liquid milk and is frequently treated as a byproduct of dairy production [12,13]. The increasing interest in the field is also reflected in the rapid expansion of the global colostrum market, which was valued at approximately USD 2.6 billion in 2019 and is projected to reach USD 4.3 billion by 2027 [14]. Colostrum-based products are increasingly being commercialized in processed forms such as powders, capsules, tablets, and fortified dairy beverages intended for human consumption [15]. Bovine colostrum is a source of immunoglobulins and growth factors, and it has been the focus of extensive research due to its immunomodulatory, antimicrobial, antioxidant, and gastrointestinal supporting properties [14,16]. Colostrum is collected under controlled conditions and subjected to processing methods such as pasteurization and drying to ensure microbiological safety and preservation of bioactive compounds [15]. In the European Union, colostrum and colostrum-based products are regulated under specific hygiene rules for foods of animal origin (Regulation (EC) No 853/2004) and must comply with established production, safety, and traceability requirements. The regulatory framework governing the import of colostrum from third countries is outlined in Regulation (EU) No 605/2010 [17]. However, despite the widespread acceptance of bovine colostrum as a relatively safe product, it is imperative to acknowledge the potential for adverse effects, including allergic reactions triggered by milk proteins, such as caseins, and IgE-mediated responses. This consideration assumes particular significance in infants with a confirmed cow’s milk allergy [18]. This surplus, which is rich in bioactive components, offers a promising opportunity for sustainable valorization through its incorporation into functional dairy products.

Whey protein concentrate (WPC) is a high-quality dairy ingredient obtained from cheese-making by-products, rich in essential amino acids and bioactive proteins such as β-lactoglobulin, α-lactalbumin, lactoferrin, and immunoglobulins. Beyond its nutritional value, WPC is widely recognized for its technological functionality, including water-binding, emulsifying, foaming, and gelling properties, which make it a valuable stabilizer in fermented dairy systems [19,20]. Experimental studies confirm that fortification with WPC enhances fermented milk texture, increases firmness and consistency, and reduces syneresis, while also supporting the viability of starter cultures during storage [21,22]. It is important to note that whey, once regarded as an environmentally burdensome effluent, is now being valorized through WPC production, thereby transforming a former waste stream into a sustainable ingredient of high nutritional and technological value [23].

Overall, the current evidence highlights the potential associated with the development of functional dairy products enriched with bioactive compounds. On the biomedical side, colostrum supplementation has been shown to improve lean body mass and support immune and gastrointestinal function, particularly under stress or high physical load [24,25]. A recent review has highlighted its potential as a nutraceutical ingredient, while also underscoring the absence of standardized approaches concerning dosage, formulation, and clinical validation [26]. From the perspective of the consumer, functional foods are generally held in higher regard than dietary supplements, as they are associated with naturalness, convenience, and integration into everyday diets. Stress relief, digestive care, and immune support have been identified as the main drivers of consumption [27,28]. In addition to nutritional and functional attributes, sensory properties such as texture, flavor, aroma and overall acceptability are critical determinants of consumer perception and market success of functional dairy products. Incorporating novel protein sources has been demonstrated to exert a substantial influence on flavor release, mouthfeel and texture formation. Consequently, this has the potential to affect consumer acceptance and purchase intentions [29,30]. Taken together, these findings emphasize the importance of designing functional dairy products that are both technologically stable and socially acceptable, positioning colostrum and WPC as complementary ingredients in creating innovations that are accepted by consumers and have health benefits that are scientifically proven. This study aimed to evaluate the effect of bovine colostrum and whey protein concentrate on the physicochemical, microbiological, functional, and sensory properties, as well as the technological stability of fermented milk beverages during refrigerated storage.

## 2. Materials and Methods

### 2.1. Materials

Colostrum bovine skimmed powder was sourced from BEMPRESA (Lublin, Poland). Whey protein concentrate (WPC), containing 80% protein, was procured from Mlekovita (Wysokie Mazowieckie, Poland). Microfiltered cow’s milk (3.5% fat) was used as the primary base for all fermented milk preparations, sourced from Piątnica Dairy Cooperative (Piątnica, Poland). Starter culture used in the experiment was YC-X16, manufactured by CHR-Hansen (Hørsholm, Denmark). Probiotic strains were obtained from HOWARU IFF Health Sciences (New York, NY, USA) (*Bifidobacterium*—4114703460; *Lactobacillus acidophilus*—4114703294). Skimmed milk powder was sourced from Mlekovita (Wysokie Mazowieckie, Poland).

### 2.2. Preparation

Four groups of fermented milk samples were prepared, with microfiltered milk combined with our additives in the following sequence:

Sample 1 (control)—5% skimmed milk powder (SMP);

Sample 2—1% colostrum, 4% SMP;

Sample 3—1% colostrum, 4% WPC;

Sample 4—1% colostrum, 1% WPC, and 3% SMP.

The selected percentages were determined based on preliminary trials and previously published studies investigating the use of colostrum or whey protein concentrate individually in fermented dairy products [31,32,33,34,35,36]. The mixtures were heated to 85 °C for 30 min, cooled to 43 °C, and inoculated with one of four microorganism combinations:

Group A—Starter culture + *Lactobacillus acidophilus;*

Group B—Starter culture + *Bifidobacterium;*

Group C—Starter culture only;

Group D—Starte culture + *Bifidobacterium* + *Lactobacillus acidophilus*.

The yogurt starter was added at 40 mg per 100 mL of milk, and probiotic cultures were supplemented at an additional 20 mg per 100 mL The fermentation process was conducted at a temperature of 43 °C, with the pH level reaching 4.7. Subsequent to the fermentation process, the yogurts were cooled to refrigeration temperature (4–6 °C) and stored for a period of two weeks.

### 2.3. pH and Acidity

The pH of the fermented milk samples was measured using a digital pH meter inoLab (WTW Wissenschaftlich-Technische Werkstätten GmbH, Weilheim, Germany), according to AOAC Official Method 973.41 [37]. Titratable acidity was determined using the Soxhlet–Henkel (°SH) method, where the samples were titrated with 0.25N NaOH in the presence of phenolphthalein as an indicator [38]. Measurements were taken at three time points: after 1, 7 and 14 day of storage.

### 2.4. Color Analysis

Color parameters (L*, a*, b*) were assessed using a Minolta colorimeter (Minolta Co., Osaka, Japan). Measurements were performed in triplicate, and results were expressed as mean ± standard deviation. Measurements were taken at three time points: after 1, 7 and 14 day of storage [39].

### 2.5. Viscosity

The viscosity of fermented milk samples was measured at room temperature using HAAKE™ MARS™ Rheometers (Thermo Fisher Scientific, Waltham, MA, USA). Measurements were conducted at a constant shear rate to ensure consistency. Measurements were taken at three time points: after 1, 7 and 14 day of storage [40].

### 2.6. Syneresis Measurement

Syneresis was assessed by centrifuging the samples at 4000 rpm for 10 min at 20 °C [41]. The amount of whey separated was measured and expressed as a percentage of the total sample weight. Measurements were taken at three time points: after 1, 7 and 14 day of storage.

### 2.7. Microbiology

Microbial counts of lactic acid bacteria and probiotic microorganisms were determined using selective culture media as follows:

- MRS Agar (Merck/Sigma-Aldrich, Darmstadt, Germany) was used for the enumeration of *Lactobacillus delbrueckii* subsp. *bulgaricus*. Plates were incubated at 37 °C for 48 h.
- M17 Agar (Merck/Sigma-Aldrich, Darmstadt, Germany) was used for the enumeration of *Streptococcus thermophilus*. Plates were incubated at 37 °C for 48 h.
- *Bifidobacterium* spp. were enumerated using Bifidus Selective Medium (BSM) supplemented with BSM Supplement (Merck/Sigma-Aldrich, Darmstadt, Germany) and incubated under anaerobic conditions at 37 °C for 48–72 h.

After incubation, colonies were counted and the results were expressed as logarithmic colony-forming units per gram of sample (log10 CFU/g). *Lactobacillus acidophilus* was included as part of the probiotic consortium; however, it was not enumerated separately due to the lack of selective differentiation from yogurt starter lactobacilli using culture media.

### 2.8. Antioxidant Activity

ABTS antioxidant capacity was measured by the reduction of ABTS radical cations, with absorbance read at 734 nm using a spectrophotometer according to a previously used method [42]. Ferric reducing antioxidant power (FRAP) was determined by the reduction of ferric ions to ferrous ions at 593 nm according to a method described previously [43]. Measurements were taken at three time points: after 1, 7 and 14 day of storage.

### 2.9. Sensory Evaluation

Sensory evaluation of samples on the first day of storage was performed using a 5-point hedonic acceptance test. Prior to the evaluation, panelists participated in two training sessions. During these sessions, they were familiarized with the evaluation procedure, sensory attributes, and the use of the 5-point hedonic scale [44]. Reference samples were discussed to ensure a common understanding of the assessed parameters. The trained panel consisted of 10 assessors (aged 23–45 years), including both male and female participants, who were regular consumers of fermented dairy products. The evaluation was conducted in individual sensory booths under controlled lighting and temperature conditions, in accordance with ISO 13299:2016 guidelines [45]. Samples were coded with random codes and presented in randomized order. Panelists evaluated samples during two separate sessions. Water was provided for palate cleansing between samples. Results were averaged and expressed as mean values. Bioethics Approval: Resolution No. N0N00000.020.1.8.4.2024 of the Rector’s Committee for Ethics in Scientific Research, Wrocław University of Environmental and Life Sciences, dated 21 October 2024.

### 2.10. Statistical Analysis

Physicochemical, antioxidant activity and microbiological analyses were conducted in triplicate (n = 3). The results were analyzed using a two-way ANOVA, followed by Tukey’s post hoc test to determine significant differences. To facilitate interpretation of product quality at each storage stage, statistical comparisons between formulations and starter culture groups were performed separately for each storage day (1, 7, and 14 days). Statistical analyses were performed using R (version 4.5.2; R Foundation for Statistical Computing, Vienna, Austria) within the RStudio 4.5.2 environment (Posit PBC, Boston, MA, USA). Initial data processing and visualization were carried out using Microsoft Excel 365 (Microsoft Corporation, Redmond, WA, USA). A significance level of *p* < 0.05 was applied.

## 3. Results and Discussion

### 3.1. Physicochemical Parameters

The findings suggest that the incorporation of colostrum and whey protein concentrate did not result in significant changes in pH during refrigerated storage. Across the full range of formulations and starter culture variants, pH values demonstrated stability over a 1, 7, and 14 day period. This finding indicates that neither of the ingredients examined caused a disruption to the acid–base balance of the fermented milk matrix (Table 1). In contrast, titratable acidity (°SH) was influenced by both the starter culture and the formulation composition. As demonstrated in Table 1, samples that underwent fermentation with starter cultures A and B exhibited consistently higher °SH values in comparison to those containing cultures C and D. Furthermore, significant disparities were identified among the various formulation variants, with elevated titratable acidity levels observed in samples comprising a greater proportion of skimmed milk powder. Formulations enriched with whey protein concentrate exhibited reduced °SH values.

Despite these differences, titratable acidity remained within a relatively narrow range throughout the storage period, indicating a controlled and stable acidification process. The simultaneous stability of pH and the moderate variation in °SH indicate a buffering effect associated with the presence of colostrum and whey proteins. This buffering effect may contribute to maintaining physicochemical stability during refrigerated storage. Sánchez-Macías et al. (2024) demonstrated that colostrum alone modulates acidity during fermentation, thereby stabilizing the physicochemical properties of the product [46]. Similarly, Gantumur et al. (2024) found that WPC fortification maintains consistent acidity and pH in low-fat yogurt due to its stabilizing impact on the protein network [32]. These observations align with the results presented here, which demonstrate the synergistic effects of colostrum and WPC in enhancing pH stability. Both acidity parameters remained stable and consistent, highlighting the stability of formulation and its resistance to variations over time and across different experimental groups.

A comparison of the L*, a*, and b* parameters revealed differences in color attributes primarily associated with formulation variants and storage time (Table 2). Over the storage period, significant changes were observed in lightness (L*) for samples 1, 2, and 3, indicating dynamic adjustments in surface or compositional properties between 1, 7, and 14 days of storage. Moreover, sample 4 demonstrated enhanced stability, exhibiting no substantial alterations in L* over the storage duration. The chromatic parameter a* demonstrated stability across all samples and storage times, indicating minimal alterations in the red-green color balance. Parameter b* exhibited minor yet significant variations between formulation variants, suggesting slight alterations in the yellow–blue component of color. However, no consistent time-dependent trends were identified. No significant differences in color parameters were detected between the groups of starter cultures, indicating that the type of starter culture did not influence the color attributes of the fermented milk beverages. The findings demonstrate that lightness (L*) is the most responsive color parameter, with formulation composition having the greatest impact, and storage time the least. Chromatic parameters exhibited relative stability under all tested conditions.

These findings are consistent with those of previous studies, which have demonstrated the influence of compositional and surface properties on lightness in dairy products [47]. Differences in specific groups in lightness, which were significant at early time points but diminished by day 14, further illustrate the role of storage in reducing variability [48]. The minimal impact of additives on color stability indicates that their low concentrations were insufficient to exert a significant influence on chromatic properties. However, the reflective properties of white dairy products, as previously reported in the literature, may introduce glare artifacts during brightness measurements, potentially affecting L* readings [49].

**Figure 1:**
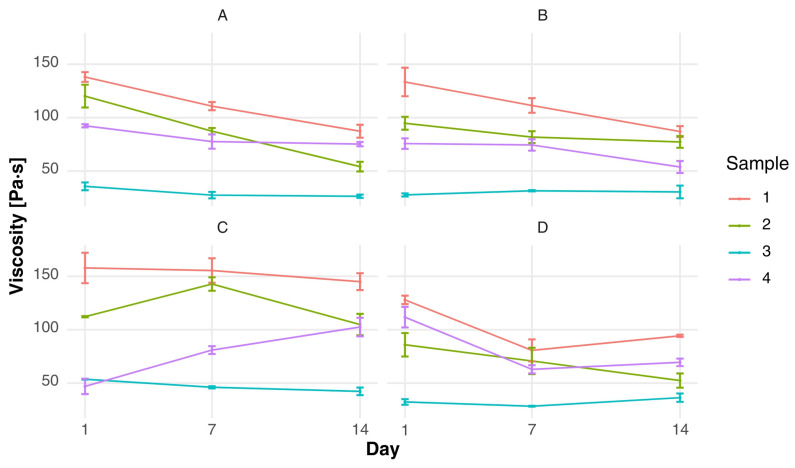
Changes over time in shear stress of fermented milk clot in four starter culture variants [Pa]: (**A**) fermented milk with a starter culture supplemented with *Lactobacillus acidophilus*; (**B**) fermented milk with a starter culture supplemented with *Bifidobacterium* spp.; (**C**) fermented milk with the starter culture alone; and (**D**) fermented milk with a starter culture supplemented with both *Bifidobacterium* spp. and *Lactobacillus acidophilus*.

**Figure 2:**
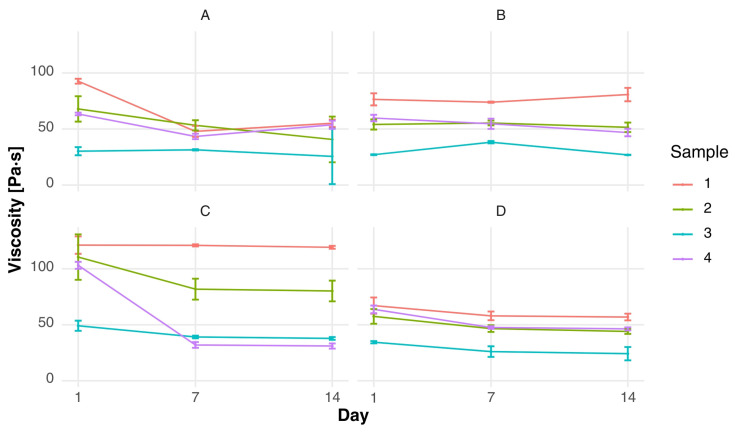
Changes over time in shear stress of mixed fermented milk in four starter culture variants [Pa]: (**A**) fermented milk with a starter culture supplemented with *Lactobacillus acidophilus*; (**B**) fermented milk with a starter culture supplemented with *Bifidobacterium* spp.; (**C**) fermented milk with the starter culture alone; and (**D**) fermented milk with a starter culture supplemented with both *Bifidobacterium* spp. and *Lactobacillus acidophilus*.

As demonstrated in Figure 1 and Figure 2, there were clear differences in shear stress (τ) and viscosity (η) observed between formulations and starter culture variants. In the context of fermented milk clots, the formulation composition has been demonstrated to have a significant impact on rheological behavior, with substantial disparities becoming apparent even after a period of one week of storage. It was found that samples containing whey protein concentrate (sample 3) consistently exhibited the lowest levels of shear stress and viscosity across all starter culture variants (e.g., τ = 35.73 ± 3.64 Pa and η = 0.35 ± 0.04 Pa·s in variant A after one week), indicating a weaker protein network compared to the control. In contrast, the control samples (sample 1) exhibited significantly higher resistance to deformation, with shear stress values exceeding 130 Pa and viscosity values above 1.3 Pa·s in several variants.

During storage, fermented milk clots showed a gradual decrease in both shear stress and viscosity, although these changes remained moderate. In the majority of variants, viscosity decreased from approximately 1.3–1.6 Pa·s after one week to values around 0.8–1.0 Pa·s after three weeks, indicating the structural stability of the gel matrix during refrigerated storage. This behavior is indicative of progressive protein rearrangement within the gel, as opposed to extensive structural breakdown, as previously reported for colostrum [50]. In the case of mixed yogurts, temporal changes in rheological properties were more pronounced. A general decline in shear stress and viscosity was observed between the first and third week of storage, particularly in formulations containing whey protein concentrate. For instance, in variant A, sample 3 showed a decrease in viscosity from 0.30 ± 0.04 to 0.13 ± 0.03 Pa·s over the storage period, suggesting increased susceptibility of the mixed matrix to enzymatic activity and structural relaxation.

It was demonstrated that sample 4, which combined whey protein concentrate and skimmed milk powder, exhibited more balanced rheological properties. For instance, in variant A, following a one week period, the values of shear stress and viscosity increased to intermediate levels (τ = 92.43 ± 1.49 Pa; η = 0.91 ± 0.01 Pa·s), thereby indicating the stabilizing effect of skimmed milk powder on the protein matrix. This finding lends further support to the hypothesis that skimmed milk powder has the capacity to neutralize the network-weakening effect of whey proteins, thereby enhancing structural integrity. The decline observed between 1 day and 14 day is attributed to enzymatic activity and structural relaxation. These changes reflect the critical influence of fermentation and matrix interactions on the structural integrity of dairy systems [21]. The initial viscosity of the fermented milk clots was higher, and this declined more significantly in the starter-only and *L. acidophilus* variants. Conversely, mixed fermented milk exhibited more stable viscosity trends, particularly in variant B and D, which suggests that these matrices have a stabilizing effect. These findings are consistent with studies on the optical and structural properties of raw milk and its derivatives, which emphasize the importance of formulation in modulating rheological behavior [49]. From a mechanistic perspective, the observed differences in viscosity and shear stress can be explained by modifications in protein–protein interactions during gel formation. The incorporation of whey protein concentrate has been demonstrated to alter the casein-to-whey protein ratio, with the potential to interfere with optimal casein micelle aggregation, particularly when the degree of whey protein denaturation is high. As demonstrated by Sodini et al. (2005) [40], yogurt fortified with specific whey protein concentrates exhibited reduced firmness and lower apparent viscosity in comparison to skim milk powder-enriched systems, particularly when elevated levels of denatured whey proteins were present. This finding lends further support to the weaker network structure observed in sample 3 [40].

The gradual decline in viscosity during storage can be attributed to continued post-acidification and limited proteolytic activity of lactic acid bacteria, leading to progressive rearrangement of protein aggregates within the gel matrix. Comparable alterations in storage-related characteristics within fortified dairy systems have been documented by Mitra et al. (2022), who emphasized that rheological behavior is influenced not just by protein concentration but also by the structural arrangement of the matrix [51].

Furthermore, the enhanced stability evidenced in formulations combining whey protein concentrate with skimmed milk powder may be attributable to elevated total solids and augmented intermolecular interactions, which facilitate water-holding capacity and gel cohesion. The synergistic effects of optimized protein systems in reinforcing yogurt texture and reducing structural weakening during storage have also been described in recent studies on polymerized whey protein systems [52].

As shown in Table 3, the data demonstrate that syneresis, indicative of whey leakage, was influenced by both the formulations and the microbial cultures. Syneresis is indicative of the water-holding capacity of the samples, with Sample 3 consistently demonstrating higher syneresis across the majority of groups particularly after 14 days. This suggests that the samples may have a reduced capacity to retain water, which may be attributed to structural rearrangements in the gel network. In contrast, samples 1 and 2 exhibited lower syneresis levels, which suggests superior structural stability and water-holding capacity. With regard to the microbial groups, in groups A and B, sample 2 consistently showed the lowest syneresis values, whereas sample 3 exhibited the highest leakage throughout storage, while group D showed more differentiated trends, with sample 1 remaining stable over time and samples 2, 3 and particularly sample 4 showing a marked increase in syneresis during storage. The findings are in accordance with those of previous studies, which indicate that the addition of WPC affects the gel structure and syneresis behavior. The addition of WPC at lower concentrations has been observed to increase brittleness and susceptibility to syneresis. This is attributed to a reduction in gel firmness and more pronounced viscous properties observed in formulations with lower WPC concentrations. An increase in WPC concentration has been observed to enhance the elastic properties of fermented milk gels, thereby reducing syneresis. This is consistent with the observation that higher casein content, which lacks strong water-binding interactions, leads to greater syneresis and lower water-holding capacity [22].

### 3.2. Functional Properties

**Figure 3:**
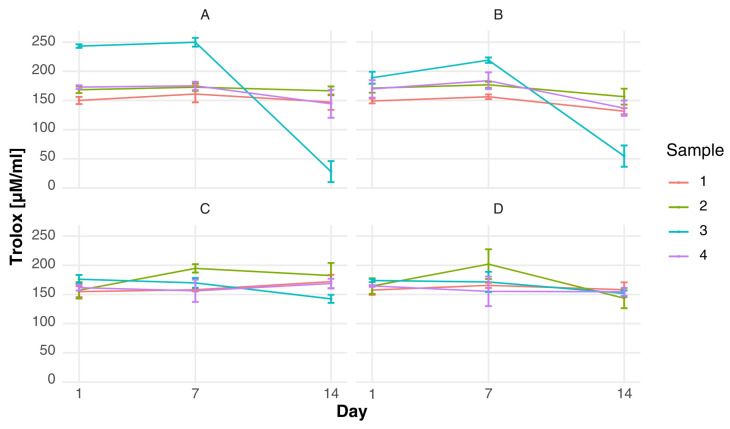
Changes over time in ABTS antioxidant activity of fermented milk in four starter culture variants [Trolox uM/mL]: (**A**) fermented milk with a starter culture supplemented with *Lactobacillus acidophilus*; (**B**) fermented milk with a starter culture supplemented with *Bifidobacterium* spp.; (**C**) fermented milk with the starter culture alone; and (**D**) fermented milk with a starter culture supplemented with both *Bifidobacterium* spp. and *Lactobacillus acidophilus*.

The antioxidant activity of the yogurt formulations, expressed as ABTS values, varied depending on formulation composition and storage time (Figure 3). Across all starter culture variants, samples containing colostrum demonstrated higher radical scavenging capacity in comparison to the control, with sample 3 exhibiting the highest ABTS activity during the initial phases of storage (e.g., 243.26 ± 3.00 µM Trolox/mL in variant A and 219.11 ± 4.60 µM Trolox/mL in variant B on day 7). In the majority of samples, an increase in ABTS activity was observed between days 1 and 7, reaching a maximum at mid-storage, followed by a decline by day 14. This tendency manifested most distinctly in colostrum-enriched formulations, where a significant decrease was observed in sample 3 (from 251.12 ± 10.19 to 28.08 ± 18.01 µM Trolox/mL in variant A, and from 219.11 ± 4.60 to 54.69 ± 18.22 µM Trolox/mL in variant B). Conversely, samples exhibiting lower levels of colostrum demonstrated more moderate alterations over time. Variation between starter culture were less noticeable than the formulation effects; however, variants C and D generally exhibited slightly lower ABTS values throughout the storage period, indicating a reduced contribution of microbial metabolism to antioxidant potential in these systems. The decline in ABTS activity observed at later storage stages may be associated with the oxidative degradation of antioxidant compounds and reduced bioavailability over time.

**Figure 4:**
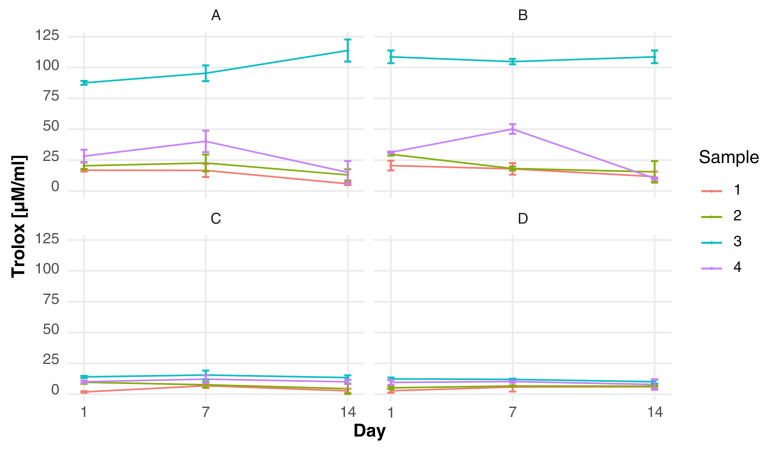
Changes over time in FRAP antioxidant activity of fermented milk in four starter culture variants [Trolox uM/mL]: (**A**) fermented milk with a starter culture supplemented with *Lactobacillus acidophilus*; (**B**) fermented milk with a starter culture supplemented with *Bifidobacterium* spp.; (**C**) fermented milk with the starter culture alone; and (**D**) fermented milk with a starter culture supplemented with both *Bifidobacterium* spp. and *Lactobacillus acidophilus*.

The FRAP assay revealed a different pattern of antioxidant behavior (Figure 4), highlighting the importance of both formulation and starter culture composition. It was evident that variants A and B exhibited significantly higher reducing capacity in comparison to variants C and D, particularly in sample 3, where a notable increase in FRAP values was observed during storage (from 87.55 ± 1.54 to 133.74 ± 18.97 µM Trolox/mL in variant A). In variant B, sample 3 exhibited consistently elevated FRAP values throughout the storage period (approximately 105–109 µM Trolox/mL). These findings are in accordance with research on colostrum’s antioxidant capacity, which identifies its phenolic compounds and bioactive peptides as the primary contributors to free radical scavenging and reducing power [9]. Furthermore, the antioxidant properties of whey protein isolates treated with advanced techniques are enhanced due to structural modifications that increase the exposure of antioxidant amino acids [53]. From a mechanistic perspective, the antioxidant activity observed in the experimental samples may be associated with the presence of bioactive compounds naturally occurring in colostrum, including lactoferrin, immunoglobulins and low-molecular-weight peptides with documented antioxidant potential [10,54,55]. During the fermentation process and subsequent storage, there is evidence to suggest that the limited proteolytic activity of lactic acid bacteria may contribute to the release of additional bioactive peptides. This, in turn, may enhance radical scavenging capacity. As posited by Mashayekh et al. (2023), analogous observations have been reported in relation to the generation of antioxidant peptides during the fermentation and storage of protein enriched dairy systems [56].

Observations made at the 7 and 14 day points following the commencement of refrigerated storage may be indicative of progressive biochemical transformations within the matrix. These transformations may include peptide release and structural modification of proteins. Comparable storage-related variations in the functional properties of colostrum-based fermented products have been described by Arkan et al. (2022) [57]. These findings suggest that moderate fluctuations in antioxidant activity represent dynamic biochemical processes within the matrix rather than functional deterioration. The results of the study demonstrate that the supplementation of colostrum has a beneficial effect on the enhancement of antioxidant potential, while ensuring the stability of the product during storage.

The microbiological results indicate that both the composition of the starter cultures and the formulation of the samples influenced the viability of *Streptococci* and *Lactobacilli* during refrigerated storage (see Table 4). The observed variations in *Streptococci* counts were associated with the type of microbial group, with neither formulation nor storage time resulting in significant alterations in their overall viability. Among the formulations, sample 2 exhibited relatively high and stable *Streptococci* counts during the initial week of storage, while sample 3 demonstrated a more pronounced decline by day 14. Starter culture variants A and B demonstrated greater stability of *Streptococci* populations compared to variants C and D, with the most evident reduction observed in group C towards the end of storage. In contrast, the viability of *Lactobacilli* was found to be contingent on both the microbial group and the formulation. The highest concentrations of *Lactobacilli* were observed in group A, particularly in sample 4, which demonstrated stability throughout the entire storage period. A decline in the overall viability of lactobacilli was observed in groups B and C, suggesting a less conducive environment for bacterial survival in these variants.

It has been demonstrated in research that the addition of whey proteins, particularly those that have undergone heat denaturation, enhances the survival of probiotics by binding water and creating a stable environment for microbial growth [11]. Furthermore, the interaction between whey proteins and colostrum bioactives has been demonstrated to enhance the growth of *Lactobacilli* and *Streptococci.* This is due to the provision of essential nutrients and the support of biofilm formation, which protects bacteria from environmental stresses [46].

**Figure 5:**
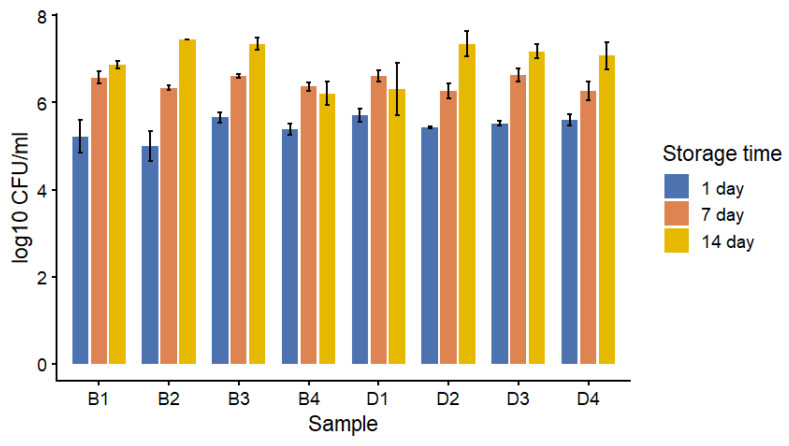
Viability of *Bifidobacterium* spp. in fermented milk samples during refrigerated storage (1, 7, and 14 days). Values are expressed as log10 CFU/g. (B) Fermented milk with a starter culture supplemented with *Bifidobacterium* spp.; (D) fermented milk with a starter culture supplemented with both *Bifidobacterium* spp. and *Lactobacillus acidophilus*.

The viability of *Bifidobacterium* spp. during refrigerated storage is presented in Figure 5. On the first day of storage, the range of *Bifidobacterium* counts in samples from group B was between 5.00 ± 0.64 and 5.66 ± 0.12 log10 CFU/g, depending on the formulation. Samples containing skimmed milk powder exclusively (sample 1) exhibited lower initial counts in comparison to formulations comprising colostrum and/or whey protein concentrate. Following a period of seven days, during which the samples were stored, an increase in the viability of *Bifidobacterium* was observed in all formulations. At this time point, samples containing colostrum combined with whey protein concentrate (samples 3 and 4) exhibited higher viable counts (6.37 ± 0.10 to 6.61 ± 0.12 log10 CFU/g) compared to samples formulated with skimmed milk powder alone (6.34 ± 0.06 log10 CFU/g). Analogous trends were observed in samples from group D, where counts ranged from 6.27 ± 0.17 to 6.63 ± 0.15 log10 CFU/g. By day 14 of storage, a further increase in *Bifidobacterium* counts was observed. The final values in group B ranged from 6.21 ± 0.27 to 7.45 ± 0.00 log10 CFU/g, while samples from group D reached levels between 6.31 ± 0.81 and 7.35 ± 0.30 log10 CFU/g. The highest final counts were observed in formulations containing colostrum in combination with whey protein concentrate (samples 2 and 3), whereas samples containing only skimmed milk powder tended to show lower values.

The results of the study indicate that both the formulation of the fermented milk and the composition of the microbial ecosystem have a significant impact on the viability of *Bifidobacterium* spp. during refrigerated storage. Samples containing colostrum and whey protein concentrate exhibited higher levels of *Bifidobacterium* than formulations based only on skimmed milk powder. This finding underscores the significance of the dairy matrix composition for probiotic survival. Whey protein concentrate has been demonstrated to enhance probiotic stability by means of improving buffering capacity and providing growth-promoting peptides and amino acids [58,59]. This observation is consistent with the enhanced viability of *Bifidobacterium* observed in samples containing whey protein, particularly after 7 and 14 days of storage. Furthermore, bovine colostrum has been shown to contain bioactive components, including lactoferrin and oligosaccharides, which may further support probiotic growth and survival [60]. The comparable viability of *Bifidobacterium* in samples fermented with *Bifidobacterium* spp. alone (group B) and in combination with *Lactobacillus acidophilus* (group D) indicates that the presence of *L. acidophilus* did not adversely affect the survival of bifidobacteria. This finding indicates that the incorporation of mixed probiotic cultures into fermented dairy products can be a successful strategy, provided that an appropriate formulation is employed.

### 3.3. Sensory Evaluation

**Figure 6:**
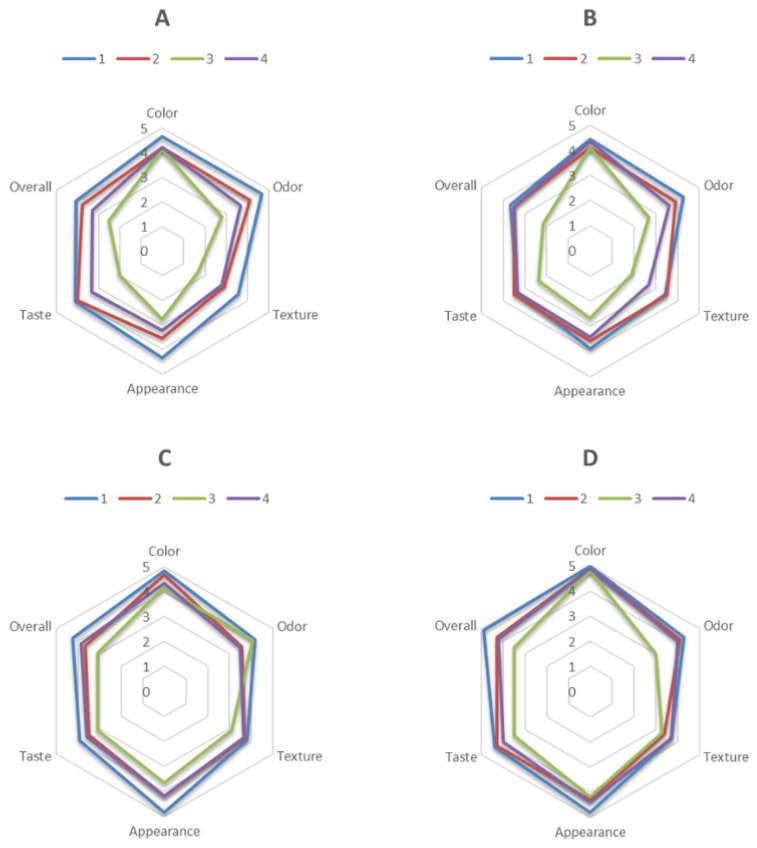
Sensory evaluation of fermented milk samples across different attributes: (**A**) fermented milk with a starter culture supplemented with *Lactobacillus acidophilus*; (**B**) fermented milk with a starter culture supplemented with *Bifidobacterium* spp.; (**C**) fermented milk with the starter culture alone; and (**D**) fermented milk with a starter culture supplemented with both *Bifidobacterium* spp. and *Lactobacillus acidophilus*.

Sensory evaluation of fermented milk samples, as illustrated in Figure 6, revealed notable discrepancies in the sensory attributes, including color, odor, texture, appearance, taste, and overall acceptability. Group D consistently achieved the highest scores for overall acceptability, with the control sample outperforming all others in terms of color (5.00 ± 0.00), appearance (4.80 ± 0.45), and overall acceptability (4.90 ± 1.24). Group C also exhibited favorable outcomes, particularly with regard to color and taste, with samples 1 and 2 receiving high ratings. These findings are in accordance with the findings of previous research, which has indicated that the fortification of whey protein concentrate (WPC) enhances the sensory properties of a product by contributing to a creamier texture, a smoother consistency, and an improved flavor [22].

In contrast, groups A and B exhibited greater variability in sensory scores, with sample 3 consistently receiving the lowest ratings, particularly for texture and appearance. This emphasizes the considerable impact of these attributes on overall acceptability. A similar trend has been observed in previous studies, indicating that lower WPC concentrations can result in brittleness and an uneven texture, whereas higher concentrations promote smoothness and cohesiveness [32].

The most variable attributes across all groups were texture and appearance, which significantly impacted sensory performance. The addition of WPC was found to enhance these sensory aspects by forming a cohesive protein network that improves creaminess and reduces roughness [22]. However, excessive fortification can result in a dense or overly firm texture, as observed in sample 3. Furthermore, the sensory acceptability of WPC-fortified yogurt is contingent upon its capacity to enhance water-holding capacity and reduce syneresis, thereby augmenting visual and tactile properties [32].

## 4. Conclusions

This study demonstrated that the addition of colostrum and whey protein concentrate did not significantly affect pH, while titratable acidity was moderately influenced by starter culture and formulation but remained within a narrow, technologically acceptable range, confirming their physicochemical stability during storage. The color parameters exhibited minimal fluctuations, with only minor deviations in lightness (L*) being observed in the initial stages, which waned over time.

The investigation established that WPC supplementation resulted in a decrease in viscosity and shear stress. The combination of WPC supplementation with skimmed milk powder balanced these effects and ensured desirable textural properties. With regard to syneresis, samples exhibiting higher levels of WPC demonstrated greater whey separation. Conversely, colostrum-enhanced water-binding capacity and improved structural stability.

Colostrum markedly enhanced antioxidant activity, particularly during the first week of storage, whereas WPC contributed to maintaining the viability of starter lactic acid bacteria, including *Streptococcus thermophilus* and *Lactobacillus delbrueckii* subsp. bulgaricus. Furthermore, the applied formulations supported the viability of *Bifidobacterium* spp. during refrigerated storage, maintaining cell counts at levels considered adequate for probiotic dairy products. These findings confirm the complementary roles of the two ingredients: colostrum as a source of bioactive compounds and WPC as a factor that promotes and stabilizes microbial survival.

Sensory evaluation demonstrated that consumer acceptance was influenced by both formulation and starter culture. Group D generally achieved the highest overall acceptability scores, while group C also demonstrated favorable sensory performance, particularly in terms of color and taste. Samples containing moderate proportions of colostrum and WPC were typically rated more favorably across several attributes. Conversely, the addition of WPC in excess (sample 3) exerted a detrimental effect on the texture and appearance of the samples, resulting in lower overall scores across the majority of the groups. The results obtained demonstrate that the sensory quality of fermented milk products is influenced by a balanced formulation and the appropriate selection of starter culture, rather than being determined by a single compositional factor. Both the starter culture groups (A–D) and the formulation variants (1–4) are explicitly presented and discussed in order to demonstrate the combined effects of microbial composition and ingredient formulation on sensory attributes.

Moreover, the synergistic use of colostrum and WPC enables the production of stable, fermented milk beverages with enhanced functional properties, supporting their potential as innovative functional dairy products. Future research could examine interactions between bovine colostrum and whey proteins, optimize their proportions, and evaluate their combined impact on probiotic viability and product stability during refrigerated storage.

## Figures and Tables

**Table 1:** Changes in pH and °SH changes over time in four starter culture variants.

Sample		1 Day		7 Day		14 Day	
		pH	°SH	pH	°SH	pH	°SH
A	1	4.49 ± 0.01 ^Aa^	28.33 ± 0.03 ^Aab^	4.55 ± 0.00 ^Aa^	25.23 ± 0.02 ^Aab^	4.52 ± 0.01 ^Aa^	25.51 ± 0.69 ^Aab^
	2	4.51 ± 0.01 ^Aa^	31.70 ± 0.10 ^Ab^	4.54 ± 0.01 ^Aa^	27.18 ± 0.75 ^Ab^	4.50 ± 0.01 ^Aa^	27.88 ± 0.18 ^Ab^
	3	4.70 ± 0.01 ^Aa^	25.11 ± 0.11 ^Aa^	4.60 ± 0.00 ^Aa^	24.94 ± 0.08 ^Aa^	4.60 ± 0.01 ^Aa^	26.44 ± 0.63 ^Aa^
	4	4.56 ± 0.00 ^Aa^	32.10 ± 0.07 ^Ab^	4.60 ± 0.00 ^Aa^	27.39 ± 0.13 ^Aab^	4.45 ± 0.01 ^Aa^	30.43 ± 0.03 ^Ab^
B	1	4.60 ± 0.00 ^Aa^	25.16 ± 0.13 ^Aab^	4.61 ± 0.01 ^Aa^	26.30 ± 0.25 ^Aab^	4.65 ± 0.01 ^Aa^	26.13 ± 0.03 ^Aab^
	2	4.60 ± 0.01 ^Aa^	26.24 ± 0.66 ^Ab^	4.63 ± 0.01 ^Aa^	23.98 ± 0.24 ^Aa^	4.66 ± 0.01 ^Aa^	25.51 ± 0.02 ^Ab^
	3	4.68 ± 0.01 ^Aa^	24.69 ± 0.11 ^Aa^	4.68 ± 0.00 ^Aa^	23.20 ± 0.59 ^Ab^	4.68 ± 0.04 ^Aa^	24.61 ± 0.08 ^Aa^
	4	4.60 ± 0.01 ^Aa^	26.94 ± 0.12 ^Ab^	4.65 ± 0.01 ^Aa^	27.63 ± 0.78 ^Aab^	4.70 ± 0.01 ^Aa^	26.41 ± 0.29 ^Ab^
C	1	4.61 ± 0.06 ^Aa^	26.91 ± 0.18 ^Bab^	4.56 ± 0.00 ^Aa^	24.76 ± 0.69 **^Ba^**	4.55 ± 0.01 ^Aa^	24.84 ± 0.69 ^Bab^
	2	4.55 ± 0.01 ^Aa^	22.24 ± 0.19 ^Bb^	4.55 ± 0.01 ^Aa^	24.41 ± 0.05 ^Bb^	4.55 ± 0.01 ^Aa^	24.48 ± 0.03 ^Bb^
	3	4.44 ± 0.03 ^Aa^	19.93 ± 0.21 ^Ba^	4.52 ± 0.04 ^Aa^	26.21 ± 0.35 ^Bab^	4.50 ± 0.01 ^Aa^	26.24 ± 0.30 ^Ba^
	4	4.70 ± 0.01 ^Aa^	25.56 ± 0.28 ^Bb^	4.61 ± 0.01 ^Aa^	24.11 ± 0.22 ^Bab^	4.60 ± 0.00 ^Aa^	24.20 ± 0.23 ^Bb^
D	1	4.57 ± 0.00 ^Aa^	24.48 ± 0.25 ^Bab^	4.59 ± 0.01 ^Aa^	24.94 ± 0.16 ^Ba^	4.53 ± 0.08^Aa^	25.03 ± 0.17 ^Bab^
	2	4.68 ± 0.00 ^Aa^	23.74 ± 0.04 ^Bb^	4.64 ± 0.01 ^Aa^	23.54 ± 0.13 ^Bb^	4.63 ± 0.04 ^Aa^	23.60 ± 0.13 ^Bab^
	3	4.73 ± 0.02 ^Aa^	26.43 ± 0.06 ^Ba^	4.61 ± 0.01 ^Aa^	23.31 ± 0.22 ^Bab^	4.61 ± 0.01 ^Aa^	23.33 ± 0.25 ^Bb^
	4	4.61 ± 0.13 ^Aa^	24.18 ± 0.08 ^Bb^	4.62 ± 0.00 ^Aa^	25.99 ± 0.02 ^Bab^	4.60 ± 0.00 ^Aa^	26.05 ± 0.03 ^Bb^

Values are mean ± SD (n = 3). Capital superscripts indicate differences between groups, while lowercase superscripts indicate differences between variants within the same group (*p* < 0.05; two-way ANOVA with Tukey’s test).

**Table 2:** Changes in color parameters of fermented milk samples during storage.

Sample		1 Day			7 Day			14 Day		
		L*	a*	b*	L*	a*	b*	L*	a*	b*
A	1	103.02 ± 5.32 ^Aab^	−0.23 ± 0.16 ^Aa^	−1.94 ± 1.26 ^Aa^	96.20 ± 1.09 ^Aab^	−0.36 ± 0.15 ^Aa^	0.04 ± 0.38 ^Aa^	98.09 ± 6.82 ^Aab^	−0.83 ± 0.56 ^Aa^	0.29 ± 1.83 ^Aa^
	2	103.74 ± 1.04 ^Ab^	−0.04 ± 0.29 ^Aa^	−2.03 ± 0.90 ^Aab^	99.40 ± 1.30 ^Ab^	−0.11 ± 0.19 ^Aa^	0.16 ± 0.30 ^Aab^	99.98 ± 4.53 ^Ab^	−0.32 ± 0.23 ^Aa^	0.59 ± 1.42 ^Aab^
	3	98.33 ± 1.64 ^Aa^	−0.02 ± 0.12 ^Aa^	−1.77 ± 0.46 ^Aa^	99.35 ± 0.54 ^Aa^	1.04 ± 0.03 ^Aa^	3.84 ± 0.08 ^Ab^	100.59 ± 2.94 ^Aa^	0.75 ± 0.09 ^Aa^	1.51 ± 0.15 ^Ab^
	4	101.26 ± 3.48 ^Ab^	−0.25 ± 0.12 ^Aa^	−0.54 ± 1.43 ^Aa^	95.57 ± 1.34 ^Ab^	−0.27 ± 0.09 ^Aa^	0.24 ± 0.43 ^Ab^	105.40 ± 2.60 ^Ab^	−0.24 ± 0.21 ^Aa^	0.95 ± 0.23 ^Ab^
B	1	105.19 ± 1.15 ^Aab^	−0.16 ± 0.09 ^Aa^	−1.18 ± 0.73 ^Aa^	104.37 ± 0.98 ^Aab^	0.30 ± 0.09 ^Aa^	2.71 ± 0.23 ^Aa^	108.21 ± 0.93 ^Aab^	−0.03 ± 0.06 ^Aa^	0.77 ± 0.80 ^Aa^
	2	104.95 ± 1.02 ^Ab^	0.18 ± 0.38 ^Aa^	−1.26 ± 1.31 ^Aab^	105.01 ± 0.92 ^Ab^	0.92 ± 0.10 ^Aa^	1.99 ± 0.59 ^Aab^	106.77 ± 2.15 ^Ab^	0.17 ± 0.05 ^Aa^	1.20 ± 0.63 ^Aab^
	3	99.21 ± 0.78 ^Aa^	0.72 ± 0.06 ^Aa^	2.04 ± 0.15 ^Aa^	100.25 ± 2.41 ^Aa^	−0.04 ± 0.58 ^Aa^	1.24 ± 1.11 ^Ab^	97.80 ± 0.29 ^Aa^	1.32 ± 0.18 ^Aa^	2.52 ± 0.68 ^Ab^
	4	99.76 ± 2.86 ^Ab^	−0.18 ± 0.23 ^Aa^	0.53 ± 0.48 ^Aa^	105.20 ± 1.08 ^Ab^	−1.11 ± 0.26 ^Aa^	−1.35 ± 0.61 ^Ab^	108.40 ± 0.66 ^Ab^	0.40 ± 0.12 ^Aa^	1.54 ± 0.54 ^Ab^
C	1	96.20 ± 1.09 ^Aab^	−0.36 ± 0.15 ^Aa^	0.04 ± 0.38 ^Aa^	105.11 ± 3.59 ^Aab^	−0.15 ± 0.20 ^Aa^	0.69 ± 0.85 ^Aa^	104.08 ± 3.73 ^Aab^	−0.05 ± 0.15 ^Aa^	0.83 ± 0.77 ^Aa^
	2	97.58 ± 99.40 ^Ab^	0.16 ± −0.11 ^Aa^	0.58 ± 0.16 ^Aab^	98.40 ± 99.37 ^Ab^	−0.73 ± −0.44 ^Aa^	4.21 ± 4.32 ^Aab^	102.50 ± 102.55 ^Ab^	−0.80 ± −0.26 ^Aa^	3.75 ± 2.33 ^Aab^
	3	93.86 ± 1.27 ^Aa^	0.21 ± 0.10 ^Aa^	0.81 ± 0.20 ^Aa^	101.80 ± 4.57 ^Aa^	0.82 ± 0.14 ^Aa^	2.57 ± 1.43 ^Ab^	100.34 ± 3.25 ^Aa^	0.09 ± 0.75 ^Aa^	3.08 ± 1.58 ^Ab^
	4	95.57 ± 1.34 ^Ab^	−0.27 ± 0.09 ^Aa^	0.24 ± 0.43 ^Aa^	104.82 ± 2.01 ^Ab^	0.32 ± 0.15 ^Aa^	2.35 ± 0.36 ^Ab^	99.65 ± 4.31 ^Ab^	0.76 ± 0.39 ^Aa^	3.04 ± 1.01 ^Ab^
D	1	100.17 ± 2.64 ^Aab^	−0.21 ± 0.04 ^Aa^	−0.38 ± 0.83 ^Aa^	102.25 ± 3.49 ^Aab^	−0.23 ± 0.19 ^Aa^	0.46 ± 1.22 ^Aa^	103.22 ± 1.93 ^Aab^	−0.05 ± 0.18 ^Aa^	0.95 ± 0.35 ^Aa^
	2	95.91 ± 2.92 ^Ab^	−0.30 ± 0.44 ^Aa^	0.08 ± 1.22 ^Aab^	103.91 ± 2.08 ^Ab^	−0.06 ± 0.21 ^Aa^	1.21 ± 0.19 ^Aab^	103.73 ± 1.57 ^Ab^	0.12 ± 0.13 ^Aa^	1.03 ± 0.57 ^Aab^
	3	98.39 ± 1.66 ^Aa^	0.49 ± 0.10 ^Aa^	1.95 ± 0.13 ^Aa^	101.81 ± 5.22 ^Aa^	0.78 ± 0.22 ^Aa^	2.04 ± 0.25 ^Ab^	99.87 ± 1.76 ^Aa^	0.83 ± 0.08 ^Aa^	2.05 ± 0.25 ^Ab^
	4	95.91 ± 2.92 ^Ab^	−0.30 ± 0.44 ^Aa^	0.08 ± 1.22 ^Aa^	107.28 ± 2.07 ^Ab^	0.13 ± 0.07 ^Aa^	1.42 ± 1.26 ^Ab^	107.22 ± 2.06 ^Ab^	0.22 ± 0.09 ^Aa^	1.73 ± 0.96 ^Ab^

Values are mean ± SD (n = 3). Capital superscripts indicate differences between groups, while lowercase superscripts indicate differences between variants within the same group (*p* < 0.05; two-way ANOVA with Tukey’s test).

**Table 3:** Changes in syneresis in yogurt samples during storage [mL].

Sample		Leakage [mL]		
		1 Day	7 Day	14 Day
A	1	5.10 ± 0.14 ^Aa^	4.70 ± 0.14 ^Aa^	4.40 ± 0.28 ^Aa^
	2	4.95 ± 0.21 ^Ab^	4.70 ± 0.14 ^Aa^	4.20 ± 0.01 ^Aa^
	3	6.95 ± 0.07 ^Ac^	6.75 ± 0.35 ^Ac^	6.60 ± 0.28 ^Ac^
	4	5.20 ± 0.01 ^Aa^	3.90 ± 0.14 ^Ab^	3.80 ± 0.01 ^Aa^
B	1	4.50 ± 0.14 ^Ba^	3.80 ± 0.01 ^Aa^	3.50 ± 0.42 ^Aa^
	2	4.25 ± 0.07 ^Ba^	3.60 ± 0.01 ^Aa^	3.25 ± 0.07 ^Aa^
	3	5.80 ± 0.28 ^Bb^	6.05 ± 0.07 ^Ab^	6.10 ± 0.14 ^Ab^
	4	4.95 ± 0.21 ^Ba^	4.10 ± 0.14 ^Aa^	4.05 ± 0.07 ^Aa^
C	1	2.15 ± 0.21 ^Ca^	3.30 ± 0.14 ^Ba^	3.60 ± 0.01 ^Ba^
	2	2.90 ± 0.14 ^Ca^	3.80 ± 0.28 ^Bb^	3.90 ± 0.14 ^Bb^
	3	4.05 ± 0.35 ^Cb^	4.95 ± 0.21 ^Bb^	5.10 ± 0.14 ^Bb^
	4	3.98 ± 0.25 ^Cb^	4.90 ± 0.14 ^Bb^	4.90 ± 0.14 ^Bb^
D	1	3.05 ± 0.07 ^Da^	3.00 ± 0.01 ^Ca^	3.10 ± 0.01 ^Ca^
	2	3.10 ± 0.14 ^Da^	3.80 ± 0.28 ^Cb^	4.05 ± 0.07 ^Cb^
	3	4.00 ± 0.01 ^Db^	4.25 ± 0.21 ^Cb^	4.30 ± 0.14 ^Cb^
	4	3.05 ± 0.07 ^Da^	3.95 ± 0.07 ^Cb^	4.10 ± 0.01 ^Cb^

Values are mean ± SD (n = 3). Capital superscripts indicate differences between groups, while lowercase superscripts indicate differences between variants within the same group (*p* < 0.05; two-way ANOVA with Tukey’s test).

**Table 4:** *Lactobacilli* and *Streptococci* viability in fermented milk samples across different groups during storage [log10CFU].

Sample		*Lactobacilli* [log10 CFU]			*Streptococci* [log10 CFU]		
		1 Day	7 Day	14 Day	1 Day	7 Day	14 Day
A	1	7.91 ± 0.05 ^Aa^	7.78 ± 0.01 ^Aa^	7.50 ± 0.04 ^Aa^	9.12 ± 0.31 ^Aa^	9.34 ± 0.08 ^Aa^	8.79 ± 1.18 ^Aa^
	2	8.04 ± 0.03 ^Ab^	8.18 ± 0.02 ^Ab^	7.91 ± 0.13 ^Ab^	9.34 ± 0.39 ^Aa^	9.47 ± 0.01 ^Aa^	8.89 ± 1.52 ^Aa^
	3	7.98 ± 0.01 ^Aa^	8.04 ± 0.05 ^Ac^	7.78 ± 0.11 ^Ab^	8.78 ± 0.35 ^Aa^	8.85 ± 0.27 ^Aa^	8.45 ± 1.63 ^Aa^
	4	8.15 ± 0.01 ^Ac^	8.29 ± 0.08 ^Ab^	8.08 ± 0.05 ^Ab^	8.94 ± 0.27 ^Aa^	9.30 ± 0.07 ^Aa^	8.83 ± 1.54 ^Aa^
B	1	5.96 ± 0.05 ^Ba^	5.30 ± 1.39 ^Ba^	5.12 ± 1.45 ^Ba^	9.04 ± 0.02 ^Aa^	9.23 ± 0.01 ^Aa^	8.70 ± 1.21 ^Aa^
	2	5.50 ± 1.16 ^Ba^	6.08 ± 0.02 ^Bb^	6.22 ± 0.06 ^Ba^	9.12 ± 0.06 ^Aa^	9.27 ± 0.28 ^Aa^	8.79 ± 1.48 ^Aa^
	3	5.29 ± 1.25 ^Ba^	5.92 ± 0.04 ^Bb^	6.15 ± 0.09 ^Ba^	8.76 ± 0.22 ^Aa^	9.01 ± 0.01 ^Aa^	8.30 ± 1.59 ^Aa^
	4	6.26 ± 0.08 ^Ba^	6.44 ± 0.05 ^Bb^	6.33 ± 0.22 ^Ba^	8.88 ± 0.26 ^Aa^	9.27 ± 0.05 ^Aa^	8.82 ± 1.37 ^Aa^
C	1	5.90 ± 0.90 ^Ba^	5.43 ± 1.17 ^Ba^	5.17 ± 1.22 ^Ba^	8.16 ± 0.01 ^Ba^	9.19 ± 0.16 ^Aa^	7.98 ± 1.13 ^Aa^
	2	5.49 ± 1.33 ^Ba^	6.20 ± 0.15 ^Ba^	6.40 ± 1.31 ^Ba^	8.23 ± 0.32 ^Ba^	9.13 ± 0.67 ^Aa^	8.30 ± 0.99 ^Aa^
	3	5.52 ± 1.14 ^Ba^	6.10 ± 0.20 ^Ba^	6.42 ± 1.23 ^Ba^	8.68 ± 0.65 ^Ba^	9.26 ± 0.06 ^Aa^	8.34 ± 1.22 ^Aa^
	4	6.23 ± 1.18 ^Ba^	6.15 ± 1.62 ^Ba^	6.01 ± 0.05 ^Ba^	8.31 ± 0.10 ^Ba^	9.14 ± 0.37 ^Aa^	8.37 ± 0.95 ^Aa^
D	1	7.46 ± 0.04 ^Ab^	7.36 ± 0.01 ^Aa^	8.17 ± 0.01 ^Aa^	9.22 ± 0.19 ^Aa^	9.64 ± 0.14 ^Aa^	8.38 ± 1.70 ^Aa^
	2	7.34 ± 0.02 ^Ab^	7.39 ± 0.02 ^Aa^	8.00 ± 0.01 ^Aa^	9.39 ± 0.01 ^Aa^	9.62 ± 0.11 ^Aa^	8.56 ± 1.44 ^Aa^
	3	7.26 ± 0.05 ^Ab^	7.30 ± 0.01 ^Ab^	7.53 ± 1.12 ^Ab^	8.32 ± 0.10 ^Aa^	9.22 ± 0.06 ^Aa^	8.39 ± 1.30 ^Aa^
	4	7.23 ± 0.07 ^Ac^	7.31 ± 0.01 ^Ab^	7.38 ± 1.10 ^Aa^	8.94 ± 0.03 ^Aa^	9.27 ± 0.14 ^Aa^	8.40 ± 1.42 ^Aa^

Values are mean ± SD (n = 3). Capital superscripts indicate differences between groups, while lowercase superscripts indicate differences between variants within the same group (*p* < 0.05; two-way ANOVA with Tukey’s test).

## Data Availability

The original contributions presented in this study are included in the article. Further inquiries can be directed to the corresponding author.
